# Synthesis and Atomic Transport of CoSn_3_ NanoIMC by In Situ TEM

**DOI:** 10.1021/acsomega.3c04762

**Published:** 2023-08-24

**Authors:** Jintao Wang, Jianqiang Wang, Luobin Zhang, Ziwen Lv, Hongtao Chen, Mingyu Li

**Affiliations:** † Department of Materials Science and Engineering, Harbin Institute of Technology (Shenzhen), Shenzhen 518055, China; ‡ State Key Lab of Advanced Solder and Joining, Harbin Institute of Technology, Harbin 150001, China; § Sauvage Laboratory for Smart Materials, Harbin Institute of Technology (Shenzhen), Shenzhen 518055, China

## Abstract

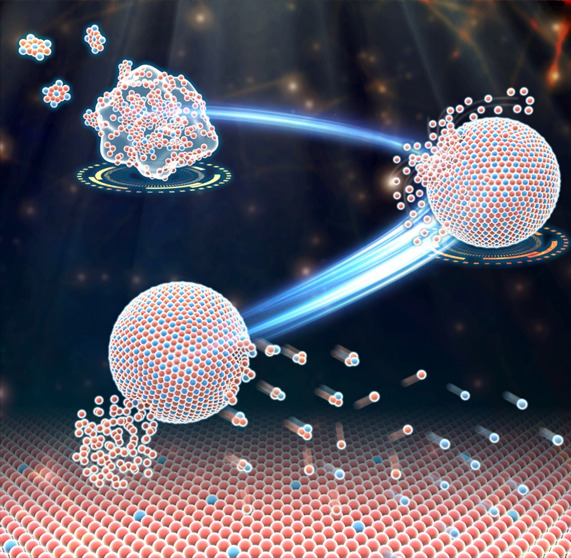

In order to optimize the interfacial properties by adding Co to the bumps of copper pillars and to overcome the strong tendency of Co to oxidize, an intermetallic compound (IMC) “capsule” was developed for the purpose of transporting elements through the intermetallic compound. In this study, we present a comprehensive analysis of the transformation process of CoSn_2_ nanoparticles into CoSn_3_ at the nanoscale using in situ heating transmission electron microscopy (TEM). The experimental results reveal that CoSn_2_ nanoparticle growth occurs through polymerization, whereas CoSn_3_ nanoparticle formation relies on the reaction between CoSn_2_ and Sn. During the initial stages of the reaction, Co dissolves and diffuses into Sn, leading to the nucleation and growth of CoSn_2_ in Sn via Ostwald ripening. As the input energy increases, vacancies in CoSn_2_ drive a reaction at the Sn/CoSn_2_ interface, resulting in the generation of CoSn_3_. In this process, Sn nanoparticles enter the CoSn_2_ structure through the “Anti Structure Bridge (ASB) mechanism” to fill vacancies. Following the codeposition process, CoSn_3_ nanoparticles were successfully plated within the Sn layer of the Cu-pillar bumps. Upon reflow heating, the CoSn_3_ nanoparticles exhibited a preference for precipitating the vacant sites within the Sn layer. This process facilitated the release of Co atoms from CoSn_2_, enabling their diffusion throughout the entire Sn layer.

## 1 Introduction

Nanocrystalline metals and IMCs have gained considerable attention in recent years, owing to their remarkable strength and unique physical properties.^1−3^ With the rapid advancement of the global semiconductor industry, the demand for integrated circuits goes beyond basic connections among a vast number of components. The industry now seeks highly integrated and reliable chip package interconnects that are systematic in nature. Vertical bonding solutions are being pursued to meet these requirements. For instance, DDR4 and wide I/O memories necessitate compact pitch I/O arrangements to achieve high bandwidth, reduced latency, and lower power consumption. However, the adoption of lead bonding has decreased due to significant issues related to parasitic capacitance. In contrast, copper pillar bumping has gained popularity due to its ability to accommodate finer pitch sizes owing to its high density and aspect ratio.^4,5^

**Figure 1:**
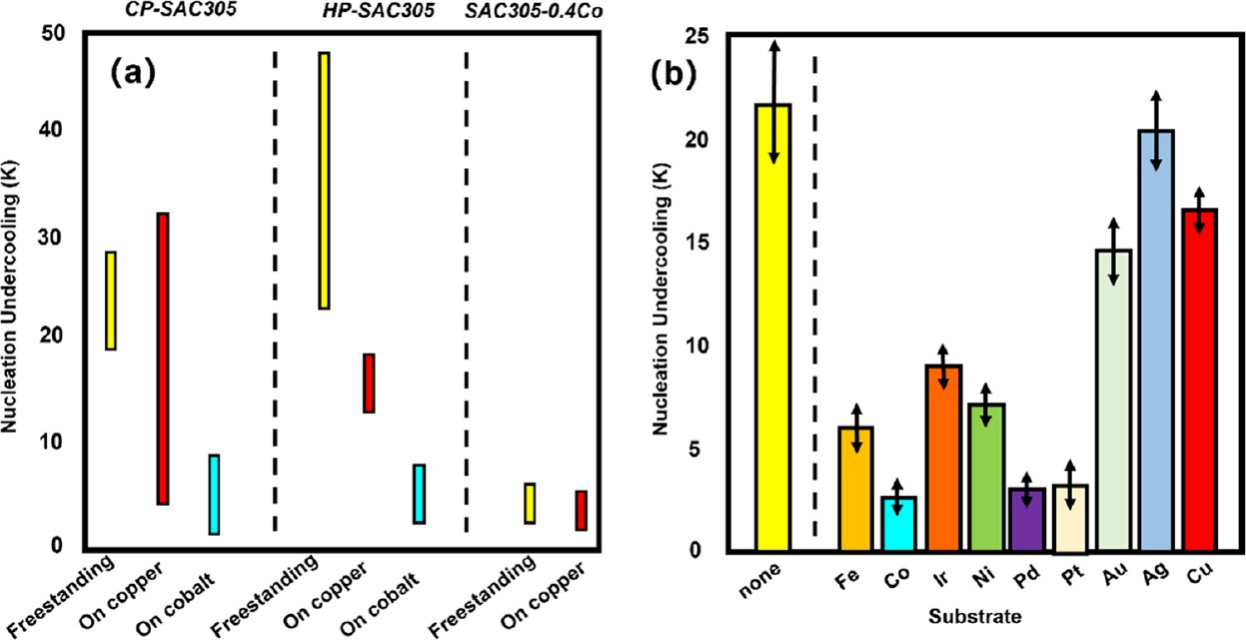
Effect of the Co substrate and Co elemental doping on the supercooling required for Sn nucleation (data from refs (5) and (6)).

Currently, copper pillar bumps are mainly interconnected with the help of the Sn3Ag plating layer. Although Sn grows in a six-fold cyclic twinning mode under sufficient subcooling conditions, Cu-pillar bumps tend to form only one large Sn grain due to the short reflow time and low reflow temperature during reflow. In many past reports, Co doping is the most effective way to reduce the supercooling degree required for Sn nucleation (Figure 1). We have also previously reported that elemental Co induces Sn to form six-fold cyclic twins (a.k.a. beach ball structures) more readily.^6^ So, we would like to realize the six-fold cyclic twinning of Sn with the help of Co doping to achieve better service reliability of Cu-pillar bumps. The addition of Co elements has emerged as an effective strategy for regulating the growth of intermetallic compounds, as demonstrated in previous studies.^6−9^ However, incorporating Co elements into copper pillar bumps presents a novel challenge, mainly due to the susceptibility of Co nanoparticles to oxidation, which can lead to the formation of CoO inclusions during storage and reflow procedures. Therefore, we need to find an intermetallic compound nanoparticle that provides Co atoms, and preferably, this compound will not become aggregately intercalated. Due to the lower pyrolysis temperature of CoSn_3_ (345 °C), CoSn_3_ nanoparticles were chosen to be the donor of Co atoms, and a new strategy was developed for the preparation of the composite plating layer. The Sn_3_Ag_0.5_Cu-CoSn_3_ composite joints were prepared by composite plating. Composite plating is a process whereby particles are fully suspended in the plating solution and deposited together with the plated material to obtain a composite coating.

**Figure 2:**
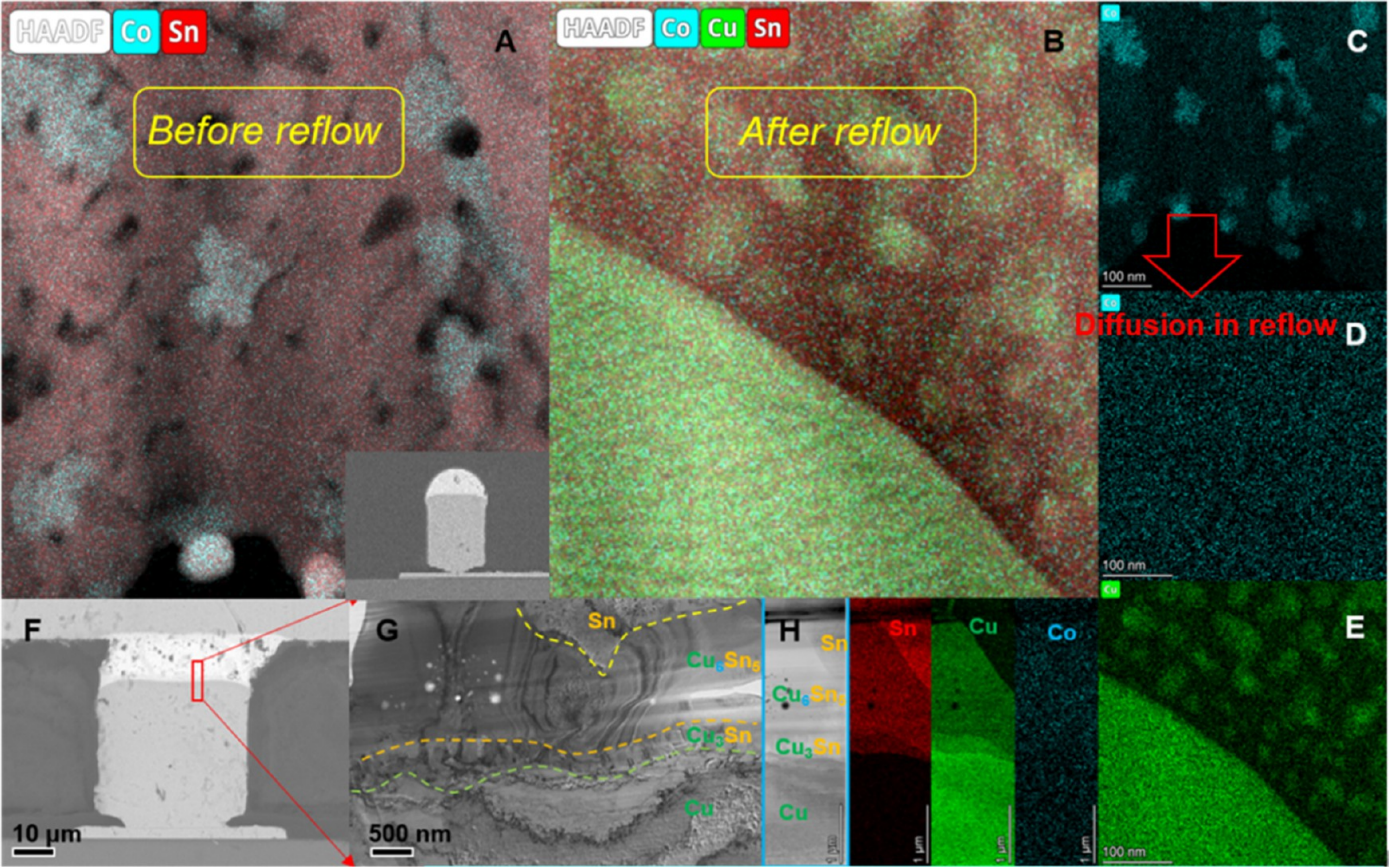
Schematic diagram of tin Co diffusion in the copper pillar bumps. (A) Energy spectrum of the Sn layer after composite deposition. (B) After reflow, the elemental distribution energy spectrum of the Sn/Cu6Sn5 interface, in which Cu atoms form atomic clusters in the tin layer and Co atoms diffuse to the whole interface. (C) Distribution of Co atoms before reflow. (D) Distribution of Co atoms after reflow. (E) Distribution of Cu atoms after reflow.

**Figure 3:**
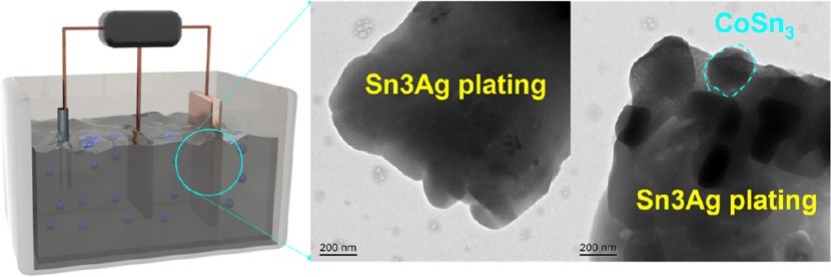
Composite plating schematic and transmission electron microscopy (TEM) images of CoSn_3_ nanocrystals into Sn_3_Ag coatings.

Peculiarly, after reflowing, the CoSn_3_ nanocrystals disappeared in the plating and the Co atoms diffused throughout the plating (Figure 2). We observed this behavior with the aid of an ambient spherical aberration electron microscope, and the atomic behavior in CoSn_3_ nanoparticles was analyzed (Figure 3).

## 2 Results and Discussion

At present, alternative strategies for producing intermetallic compounds at lower temperatures are becoming increasingly popular. In this study, we employed a “beaker chemistry”^10^ method to synthesize CoSn_2_ and CoSn_3_ nanoparticles by reducing Co and Sn precursors in an ethanol solution of TEG, followed by modulating the controlled diffusion of the precursor through layered elements at low annealing temperature (350 °C). This synthesis approach minimizes diffusion length and increases reactivity, promoting exploratory synthesis, stabilizing low-temperature phases, and allowing dynamic control of phase formation by applying ultrasound.

The ethanol solution of SnCl_2_ and CoCl_2_ was mixed in an ultrasonic environment that the atomic ratio of Co to Sn is 1:3.5 and then dropped dropwise into the ethanol solution of TEG, resulting in many black nanocrystal precipitates. Centrifuge to obtain nanocrystalline precipitates, disperse them in ethanol solution, and drop them onto the heating chip. The heating bracket is inserted into a high vacuum chamber, and in situ heating and observation are carried out using Titan ETEM G2 (FEI) spherically corrected transmission electron microscopy. Microzone heating is achieved through laser focusing, with negligible heating rates, and is maintained for 300 s at 50, 500, and 800 °C, respectively. We then deposited CoSn_3_ nanocrystals onto the Sn layer of the Cu-pillar bumps by codeposition at a voltage of 6 V. To prepare the composite plating solution, we selected a methane-sulfonic acid tin plating solution, which consisted of CH_3_SO_3_H, Sn(CH_3_SO_3_)_2_, AgCH_3_SO_3_, C_2_H_6_S_2_, and C_5_H_11_N_5_S. In addition, CoSn_3_ nanoparticles and a dispersant, poly(ethylene glycol), were incorporated into the composite plating solution. By introducing cysteine into the system, we aimed to refine the grain structure by influencing the surface double-layer configuration and electrode kinetics. Notably, the nanoparticles adsorb onto the high-energy surface regions, effectively inhibiting the growth of the most active Sn sites. The Cu-pillar bumps were then reflow soldered to the Cu substrate at a reflow temperature of 250 °C and a reflow time of 180 s. The interface before and after reflowing was then FIB cut and observed by transmission electron microscopy.

To summarize, Co^2+^ and Sn^2+^ are first reduced to particles of Co and Sn by NaBH_4_. The reaction equation is shown as follows
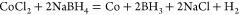

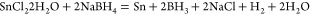
During the reaction, the reaction that produces Sn particles will proceed preferentially because the standard electrode potential of Sn^2+^/Sn in solution is −0.14 V, which is higher than the standard electrode potential of Co^2+^/Co (−0.28 V). Cobalt is a fast-diffusing element in Sn because Sn is generated before Co during the reaction, so simple Co–Sn compounds are distributed in Sn after the reduction reaction. The energy provided at room temperature is far from the activation energy required for the reaction between Co and Sn to form CoSn_3_, so external input energy is required to synthesize CoSn_3_ NPs. The tube furnace can provide energy quickly.

**Figure 4:**
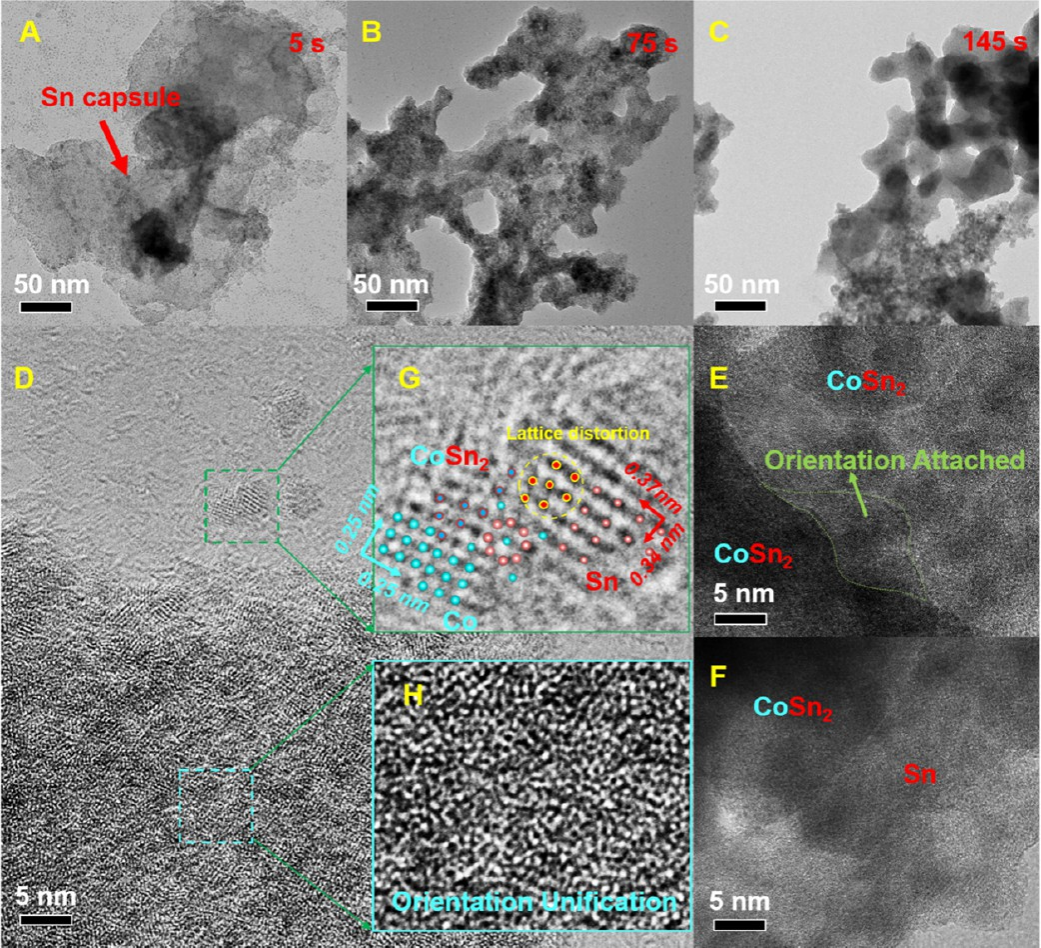
In situ observation of nucleation and growth of Co–Sn atomic clusters at 50 °C (A) 5 s, Co and Sn nucleate on an intricate network of organic compounds. (B) 75 s, Co–Sn atomic clusters merge and grow with each other. (C) 145 s, CoSn_2_ nanocrystals and surrounding Sn. (D, G) 5 s, CoSn_2_ nucleation in Co–Sn clusters (E) 75 s, CoSn_2_ nanocrystals grown through “unified orientation” (F) 145 s, Interface between CoSn_2_ and Sn (H) CoSn_2_ nanocrystals grown through uniform orientation have numerous defects such as dislocations and atomic voids.

The reduced Co and Sn particles tend to crystallize at a scale of approximately 2 nm, and most of the Co and Sn nucleate on an intricate network of organic compounds (Figure 4). This phenomenon can be attributed to the presence of functional groups on the surface of the organic compounds, which can act as sites for metal nucleation. Owing to the thermal agitation of the particles, they tend to collide and merge with each other to form the primary intermetallic compound CoSn_2_. From a more fundamental point of view, the agglomeration of nanoparticles may be triggered by a variety of factors, such as van der Waals attraction, charge–charge interactions, and dipole interactions, at a critical distance of <1 nm, below which particles rapidly agglomerate.

**Figure 5:**
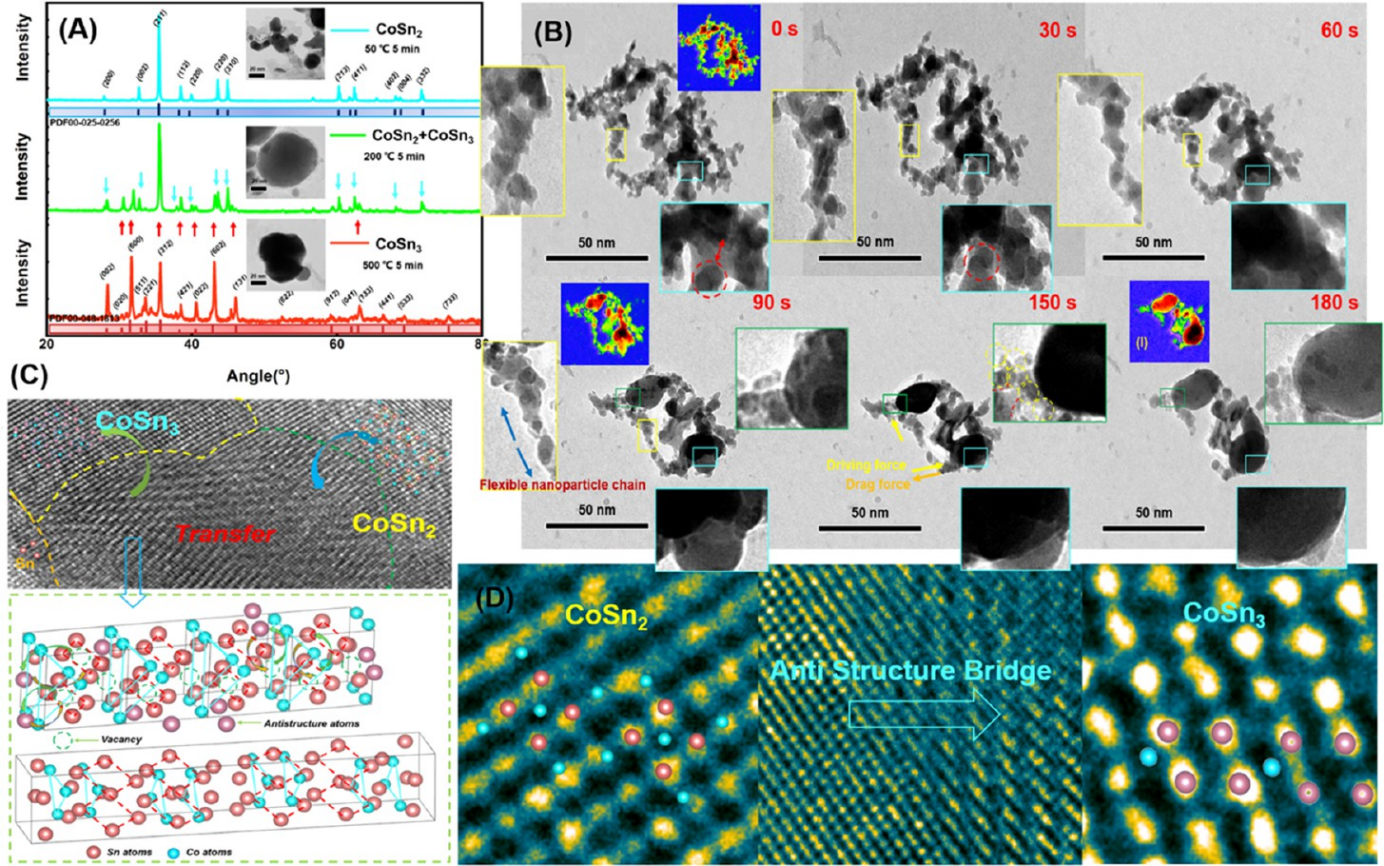
(A) XRD results of centrifuged products at different stages during the heating process. (B) In situ observation of CoSn_2_ and liquid-phase Sn mixture during the heating process in vacuum environment at 500 °C. (C) High-resolution TEM images (HRTEM) of the transition from CoSn_2_ to CoSn_3_ (at 500 °C) and the diagram of the ASB mechanism. (D) Pseudo-color image of the transition from CoSn_2_ to CoSn_3_ (at 500 °C).

However, at low temperatures, it seems that CoSn and Co_3_Sn_2_ fail to form, and X-ray diffraction (XRD, Figure 5A) analysis of the crystalline products revealed the presence of CoSn_2_ and CoSn_3_ only. During the initial stage of heating, Co–Sn clusters in the core of the organic network come together, and after the process of “orientation unification,” a primary intermetallic compound CoSn_2_ with numerous dislocations and vacancy defects is formed. Afterward, the Co–Sn clusters and Sn clusters around the organic network enter the CoSn_2_ crystal via “orientation attached” (Figure 4E,H).

The combination of two particles of equal size represents the energetically least favorable scenario (Figure 4G). The presence of nanostructures induces a high density of grain boundaries, leading to a significant phonon scattering effect, surpassing the scattering caused by carriers. Consequently, the two Co–Sn clusters rapidly bond with each other during the heating process, with their overlapping area accounting for more than half of their cross-section. As the two particles merge, they exhibit Moore’s stripe patterns, and the density of these patterns diminishes with prolonged heating, indicating a reduction in orientation errors due to crystal structure transformation.

In cases where the contact area between the two particles is less than 50% of the cross-sectional area of the smaller particle and a substantial difference in their original orientations exists, orientation additions (OA) may occur (Figure 4E). This phenomenon involves the enlargement of the center particle by consuming the surrounding smaller particles. However, due to insufficient time and energy to adjust the crystal structure, dislocations arise on the inner face.

It is noteworthy that at 50 °C, a considerable number of Sn clusters are unable to enter the CoSn_2_ crystal. After being subjected to 170 s of heating at 50 °C, a well-defined boundary between CoSn_2_ and Sn emerges (Figure 4F). Afterward, we observed the interface between CoSn_2_ and Sn under heating conditions of 500 °C.

### 2.1 Transformation

The process of CoSn_2_ reacting with molten Sn to form CoSn_3_ nanoparticles is triggered when the microzone temperature reaches 500 °C (Figure 5). The nanoparticles, due to their dipole interaction, form flexible nanoparticle chains. The initial colloid of small primary nanoparticles is highly unstable and undergoes rapid aggregation to attain size-stable aggregates. The number of growth aggregates is determined by this primary aggregation process. By adding primary nanoparticles to these larger and more stable aggregates, subsequent growth occurs. It is important to note that the growth of nanocrystals through aggregation and coalescence is a discontinuous process. This growth leads to a gradual increase in size as nanocrystals of similar size aggregate over a short period of time. The particles formed through aggregation subsequently rearrange themselves in morphology and ultimately reach a nearly spherical shape. Following aggregation, the nanocrystals gradually reshape and evolve into cross sections, presenting a single crystal or twin structure. This observation indicates that the nanoparticles undergo structural transformation during their growth process.

The process under investigation involved the formation of CoSn_2_ nanoparticles, followed by their transformation into CoSn_3_ nanoparticles because of the infiltration of Sn atoms from liquid Sn. The resulting nanoparticles were characterized using TEM and XRD testing (Figure 5). The TEM analysis revealed that a uniform and highly crystalline tetragonal CoSn_2_ NC was formed initially, with a size of approximately 10 nm. The XRD analysis confirmed the formation of tetragonal CoSn_2_ nanoparticles, with a space group *I*4/*mcm* and lattice parameters of *a* = *b* = 6.363 Å and *c* = 5.456 Å, corresponding to JCPDS No. 25-0256. During the dynamic process at 500 °C, the Sn atoms infiltrated the CoSn_2_ lattice resulting in the transformation of the tetragonal CoSn_2_ NC into CoSn_3_ NC. The resulting CoSn_3_ NC were characterized using TEM, and it was found that the transformation proceeded from the surface of CoSn_2_ nanoparticles to the center while maintaining the orientation relationship between the cube and the cube (Figure 3B). The resulting CoSn_3_ NC had a space group I41 and lattice parameters of *a* = *b* = 6.275 Å and *c* = 3.374 Å, corresponding to JCPDS No. 48-1813.

**Figure 6:**
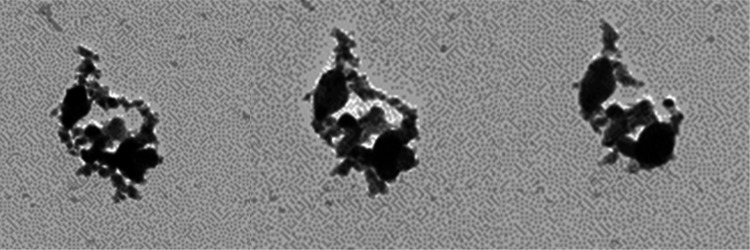
Motion picture of the CoSn_3_ formation process (GIF format. A clearer version of this we have added in the Supporting Information).

The interface between CoSn_2_ and Sn during the dynamic process showed numerous vacancies, and the formation of dislocations facilitated the transformation (Figure 3I). The growth of any IMC at the interface between solid metal and liquid solder is a result of the overall reverse diffusion of components, followed by chemical reactions between diffusion atoms. The introduction of high-density point defects at the interface not only makes the crystal structure unstable but also enhances atomic diffusion, thereby inducing rapid crystallization of CoSn_3_. By increasing the activity of point defects, the free energy of the system becomes the driving force for the formation of non-equilibrium phases. Therefore, in just a few seconds, CoSn_2_ completed its transition to CoSn_3_ (Figure 6).

The transformation from CoSn_2_ NC to CoSn_3_ NC occurred at a temperature of 500 °C, and there was no undercooling-induced phase change driving force during the process. Instead, the transformation was driven by vacancy diffusion, a phenomenon that has been extensively studied in materials science. By analyzing high-resolution images, we observed the lattice transition taking place on the (0 0 2) plane (Figure 5C, D), where Sn atoms from liquid Sn replaced vacancies. The Sn atoms changed their positions, while the Co atoms did not alter their relative positions, which suggested that the Sn atoms replaced the vacancies through diffusion within the CoSn_2_ lattice.

**Figure 7:**
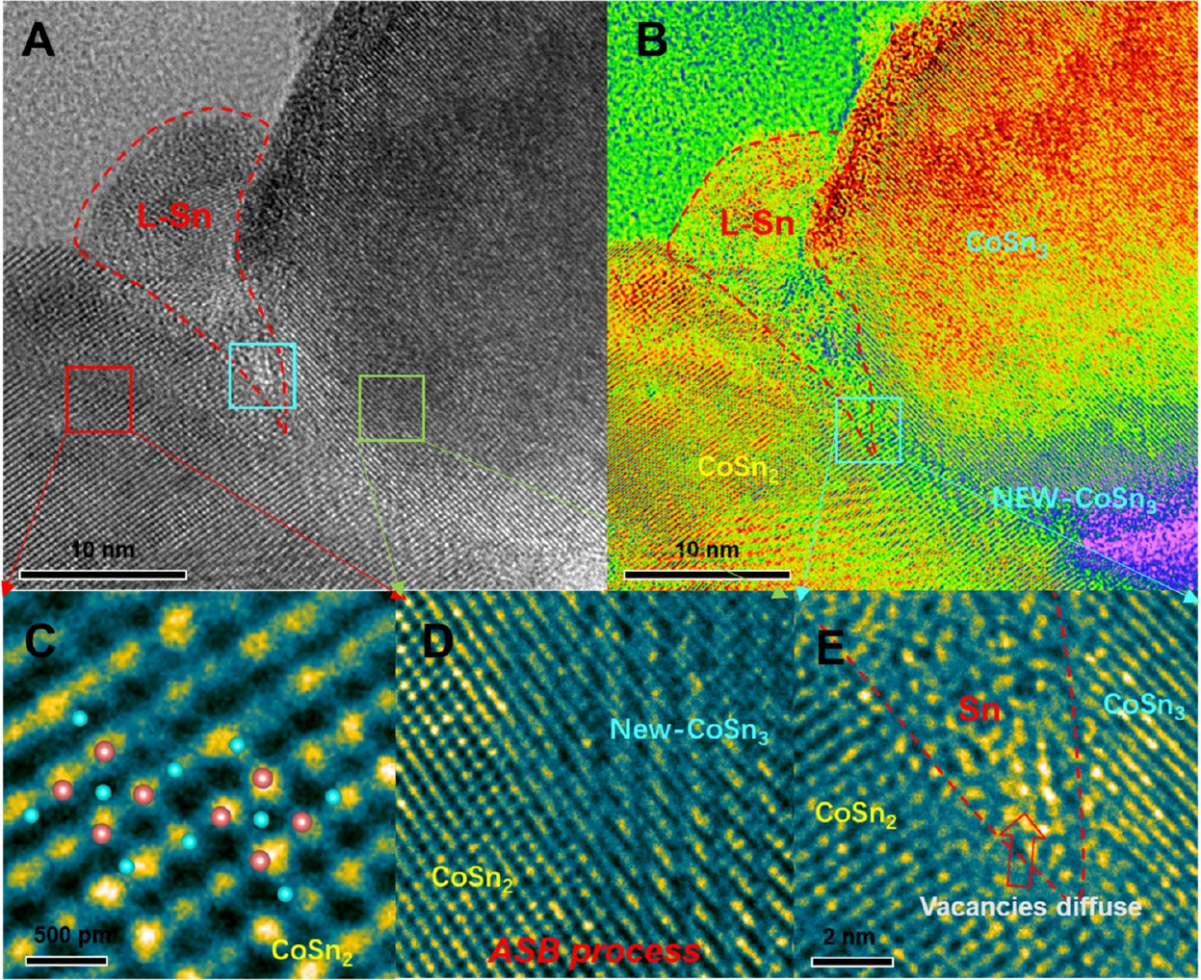
High-resolution images (HRTEM) of the transition from CoSn_2_ to CoSn_3_. (A) High-resolution images. (B) Pseudo-color image of the CoSn_3_/CoSn_2_ interface. (C) High-resolution images of the CoSn_2_ lattice. (D) Schematic diagram of the ANTI Structure Bridge (ASB) mechanism for the transition from CoSn_2_ to CoSn_3_. (E) Pseudo-color image of the CoSn_3_ lattice.

The mechanism behind this vacancy diffusion-driven phase transition can be explained by the ASB mechanism (Figure 7). According to this model, vacancies in the crystal lattice are responsible for the formation of ASB,^10−15^ which can provide low-energy diffusion pathways for atoms to migrate through the crystal lattice. During the process, Sn atoms from the liquid Sn infiltrate into the CoSn_2_ lattice by diffusing through the ASB, replacing the vacancies in the process. The ASB mechanism provides a low-energy pathway for Sn atoms to infiltrate into the CoSn_2_ lattice and for the vacancy diffusion to occur, thus driving the transformation from CoSn_2_ NC to CoSn_3_ NC. Once a vacancy is created at any site in the diffusion zone and another vacancy forms in its neighborhood before the previous vacancy is consumed by the counterflow of atoms, an instantaneous localized enhancement of vacancy concentration occurs.

It is worth highlighting that the contribution of the ASB mechanism has a percolation effect, which means that long-range diffusion through the ASB mechanism only occurs when the concentration of antistructure atoms is high enough. The premise of this mechanism is that liquid Sn brings antistructure Sn atoms to the CoSn_2_ crystals, providing a channel for their self-diffusion through vacancy migration (Figure 7). This observation is consistent with the experimental results, where Sn atoms infiltrated the CoSn_2_ lattice and replaced vacancies to form CoSn_3_ NC.

**Figure 8:**
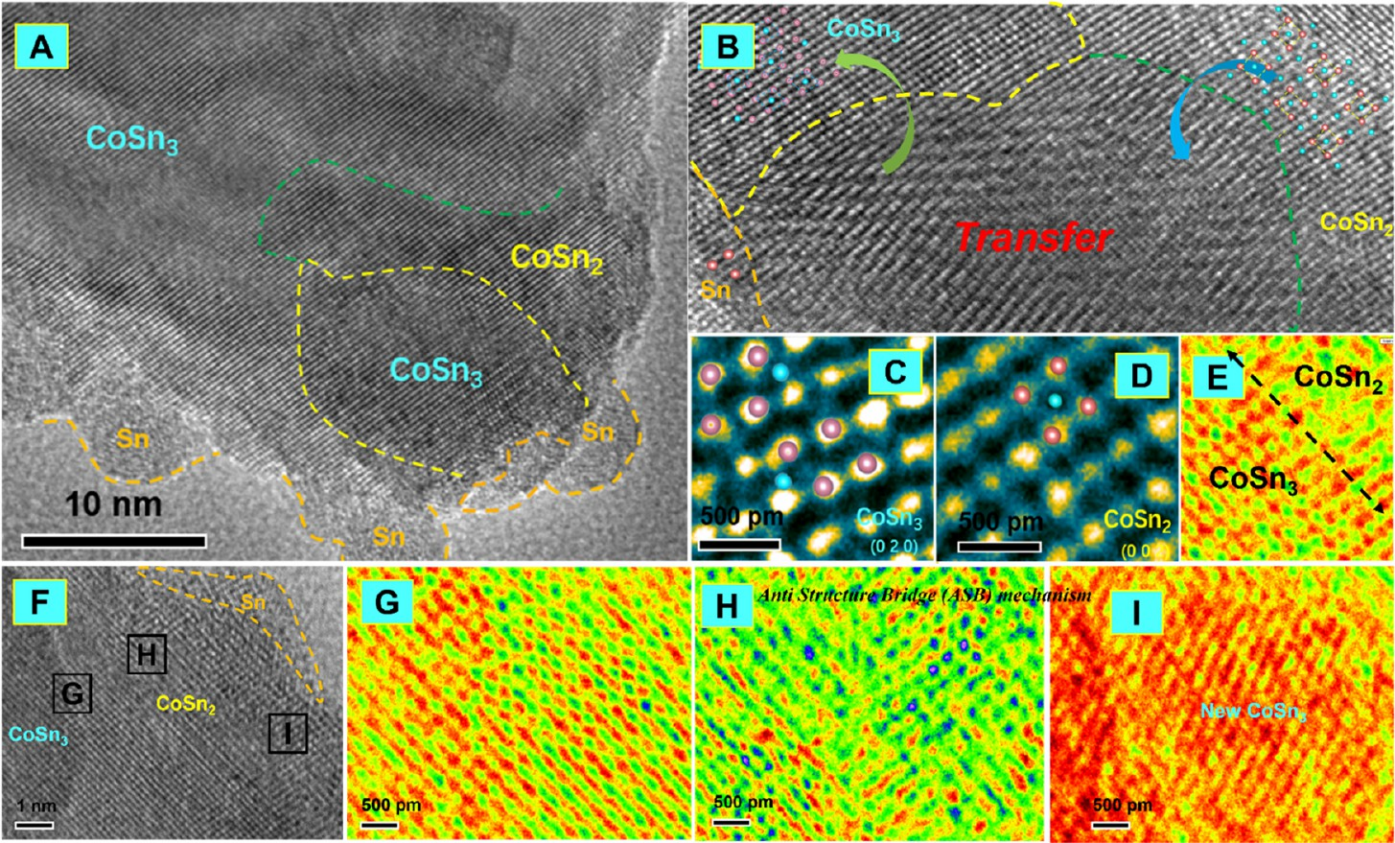
High-resolution images (HRTEM) of the transition from CoSn_2_ to CoSn_3_. (A) High-resolution images. (B) Schematic representation of intermetallic compounds for atomic transport. (C) High-resolution images of the CoSn_3_ lattice. (D) High-resolution images of the CoSn_2_ lattice. (E) Pseudo-color image of the CoSn_3_/CoSn_2_ interface. (F) High-resolution images of the CoSn_3_/CoSn_2_ interface. (G) Pseudo-color image of the CoSn_3_/CoSn_2_ interface. (H) Pseudo-color image of the CoSn_3_/CoSn_2_ interface. (I) Pseudo-color image of the CoSn_3_/CoSn_2_ interface.

As shown in Figure 8, the CoSn_2_ sublattice contains vacancies and antistructure Sn atoms. From an energy perspective, vacancies are more likely to exchange positions with antistructure Sn atoms through next-nearest-neighbor hopping compared to exchanging positions with conventional Sn atoms in one’s own lattice through nearest-neighbor hopping. When antistructure atoms are present in the adjacent lattice at the nearest or next-nearest-neighbor position, vacancies may continue to repeat this process. If the concentration of antistructure Sn atoms is high enough, they can serve as bridges for the free migration of vacancies without altering the degree of order.

In the ASB process, the diffusion flow of Co and Sn atoms is independent of each other, and their migration occurs in an acyclic manner. This mechanism provides a pathway for Sn atoms to infiltrate the CoSn_2_ lattice and drive the transformation to CoSn_3_ NC, as observed in the experiment. It is interesting to note that when the atomic ratio of Sn to Co is less than 3:1, CoSn_3_ NC will never appear, even locally. This result is consistent with the percolation effect of the ASB mechanism, which requires a sufficient concentration of antistructure atoms to enable long-range diffusion.

### 2.2 Co Atomic Transport

**Figure 9:**
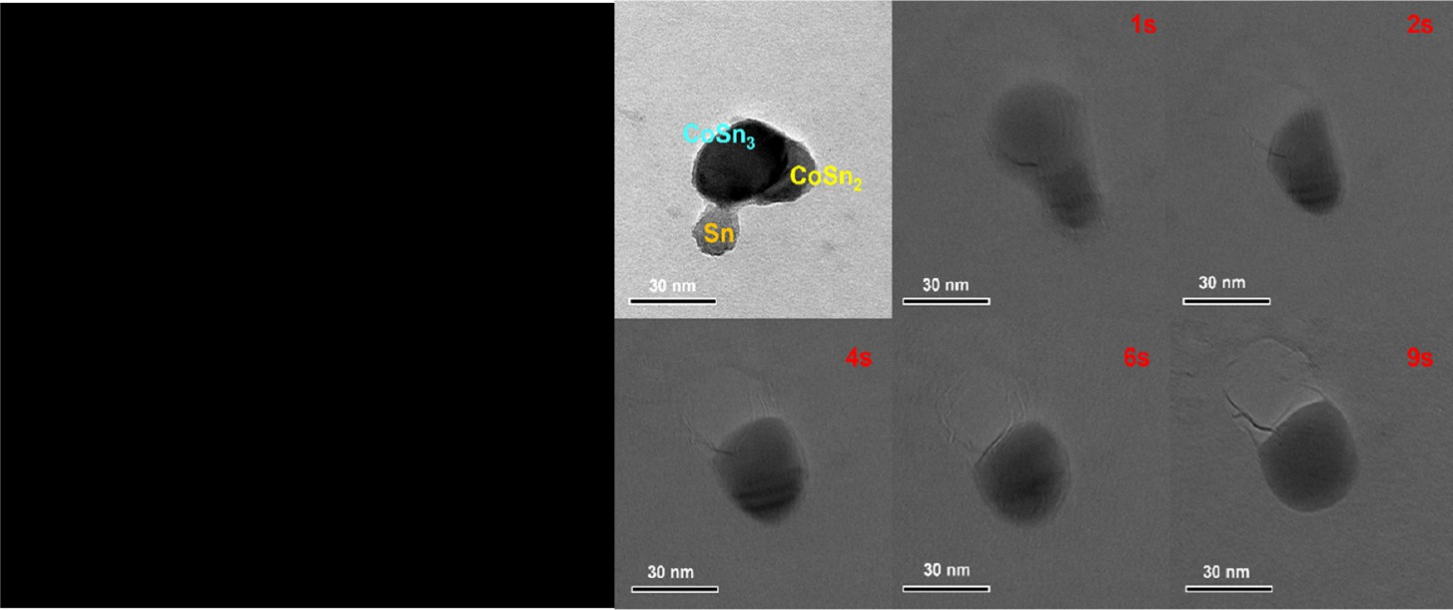
Motion picture of the CoSn_2_ dissolving directly in liquid Sn (GIF format).

**Figure 10:**
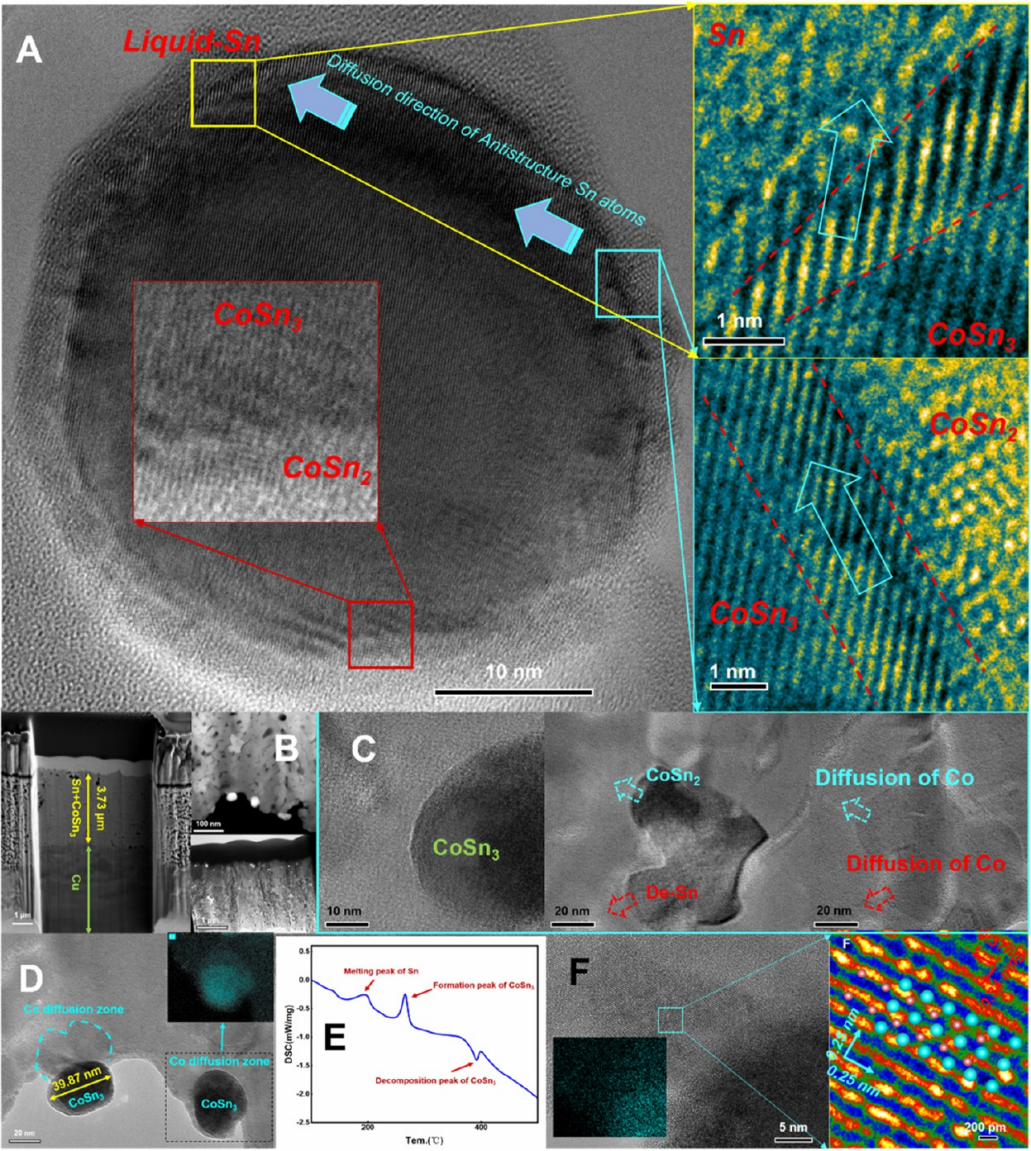
TEM images of Co atoms transported by CoSn_3_ Nps. (A) Transport channels for Co atoms in liquid Sn. (B) Composite plating. (C) Diffusion of Co atoms in Sn. (D) EDX results for diffusion of Co atoms in Sn. (E) DSC results of diffusion of Co atoms in Sn. (F) High-resolution images of the diffusion of Co atoms in Sn.

To further investigate the structural changes of CoSn_3_ under heat during the reflux process, after the synthesized particles were cooled down, we further heated them up and conducted in situ experiments using TEM. As a result, we found that a large amount of liquid-phase Sn separated from CoSn_3_ after heating at 350 °C for 2 min. In a very short time afterward, CoSn_2_ also dissolved in liquid Sn. Interestingly, during this process, CoSn_3_ did not dissolve directly but degraded into CoSn_2_ and Sn, followed by diffusion of Co atoms in the liquid Sn. This process proves that the conversion of CoSn_3_ to CoSn_2_ is reversible. The energy input during this process selectively destroys the extruded tin lattice, releasing the tin atoms. CoSn_2_ does not continue to degrade but dissolves directly in liquid Sn (Figures 9 and 10).

It is worth noting that this structural change is not entirely surprising. The crystal structure of CoSn_3_ is less stable than that of CoSn_2_. The degradation process of CoSn_3_ to CoSn_2_ and Sn is attributed to the release of excess energy to the surrounding environment. The energy released during the process of converting CoSn_3_ to CoSn_2_ is used to destroy the squeezed Sn lattice and release Sn atoms. This process confirms the metastability of CoSn_3_ in the presence of liquid-phase Sn. That is to say, the ASB mechanism is reversible. We have used this feature in the field of electronic packaging by codeposition to create CoSn_3_ Cu-pillar bumps. After reflow at 250 °C, Co diffuses from the CoSn_3_ nanocrystals across the interface and the CoSn_3_ nanoparticles disappear and are replaced by Cu_6_Sn_5_ nanoparticles (the Co element reduces the Cu_6_Sn_5_ nucleation and promotes Cu_6_Sn_5_ nucleation, Figure 2).

When CoSn_3_ is subjected to heat, due to the inverse ASB mechanism, CoSn_3_ will preferentially de-Sn (Figure 4J), and CoSn_3_ decomposes into Sn and CoSn_2_. In the presence of liquid Sn, CoSn_2_ exhibits a lattice structure that bears resemblance to Sn. Leveraging the principle of similar solubility, CoSn_2_ gradually dissolves in the liquid Sn, leading to the loss of polarity in the covalent bond. Consequently, Co atoms diffuse into the liquid Sn phase. Given the relatively large crystal spacing of liquid Sn atoms, Co atoms demonstrate rapid diffusion throughout the interface. After our tests, the CoSn3-codeposited copper pillar bumps can achieve a shear strength of over 50 MPa and a microhardness of 21 HV, which is higher than the conventional Sn–Ag copper pillar bumps.

## 3 Conclusions

**Figure 11:**
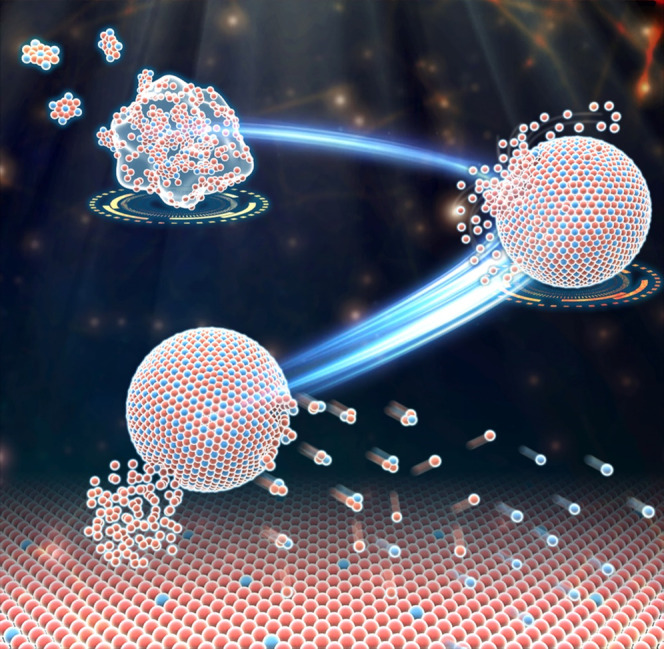
Schematic image of the formation and decomposition for the CoSn_3_ Np.

In this study, we employed the “beaker chemistry” method to synthesize CoSn_3_ nanocrystals, which were subsequently utilized in Sn3Ag plating for electronic packaging through the composite plating method. To investigate the CoSn_3_ nanocrystals and their synthesis process, we employed ambient spherical aberration electron microscopy, enabling in situ observations and insights into the Co atom transport mechanism within the composite plating layer. Upon reduction, Co–Sn nanoclusters underwent a thermal collision process on the organic matter network sites, resulting in the formation of CoSn_2_ nanocrystals. This process involved two distinct stages: orientation unification and orientation attachment (Figure 11). During the growth of CoSn_2_ nanocrystals, liquid Sn infiltrated via antistructure atomic bridges, leading to the subsequent evolution of CoSn_2_ nanocrystals into CoSn_3_ nanocrystals through the “ASB” mechanism. Under the influence of heat within the liquid Sn environment, CoSn_3_ nanocrystals preferentially decomposed into Sn and CoSn_2_ at 350 °C. Upon reaching 350 °C, Sn atoms preferentially exited the CoSn_3_ nanocrystals through antistructure bridges, and subsequently, the CoSn_2_ nanocrystals gradually dissolved in liquid Sn. This dissolution process facilitated the diffusion of Co atoms throughout the entire Sn layer.

## Supporting Information Available

CoSn_3_ formation process (also shown in Figure 6) (MP4)
